# Planning for success: overcoming challenges to recruitment and conduct of an open-label emergency department–led paediatric trial

**DOI:** 10.1136/emermed-2020-209487

**Published:** 2020-10-13

**Authors:** Louise Roper, Mark D Lyttle, Carrol Gamble, Amy Humphreys, Shrouk Messahel, Elizabeth D Lee, Joanne Noblet, Helen Hickey, Naomi Rainford, Anand Iyer, Richard Appleton, Kerry Woolfall

**Affiliations:** 1 Institute of Population Health & Society, University of Liverpool, Liverpool, UK; 2 Emergency Department, Bristol Royal Children's Hospital, Bristol, UK; 3 Faculty of Health and Applied Sciences, University of the West of England, Bristol, UK; 4 Liverpool Clinical Trials Centre, University of Liverpool, Liverpool, UK; 5 Emergency Department, Alder Hey Children's NHS Foundation Trust, Liverpool, Merseyside, UK; 6 Department of Neurology, Alder Hey Children's NHS Foundation Trust, Liverpool, Merseyside, UK

**Keywords:** epilepsy, paediatric emergency med, qualitative research, research, clinical, research, operational

## Abstract

**Background:**

Key challenges to the successful conduct of The Emergency treatment with Levetiracetam or Phenytoin in Status Epilepticus in children (EcLiPSE) trial were identified at the pre-trial stage. These included practitioner anxieties about conducting research without prior consent (RWPC), inexperience in conducting an ED-led trial and use of a medication that was not usual ED practice. As part of an embedded study, we explored parent and practitioner experiences of recruitment, RWPC and conduct of the trial to inform the design and conduct of future ED-led trials.

**Methods:**

A mixed-methods study within a trial involving (1) questionnaires and interviews with parents of randomised children, (2) interviews and focus groups with EcLiPSE practitioners and (3) audio-recorded trial discussions. We analysed data using thematic analysis and descriptive statistics as appropriate.

**Results:**

A total of 143 parents (93 mothers, 39 fathers, 11 missing information) of randomised children completed a questionnaire and 30 (25 mothers, 5 fathers) were interviewed. We analysed 76 recorded trial recruitment discussions. Ten practitioners (4 medical, 6 nursing) were interviewed, 36 (16 medical, 20 nursing) participated in one of six focus groups. Challenges to the success of the trial were addressed by having a clinically relevant research question, pragmatic trial design, parent and practitioner support for EcLiPSE recruitment and research without prior consent processes, and practitioner motivation and strong leadership. Lack of leadership negatively affected practitioner engagement and recruitment. EcLiPSE completed on time, achieving its required sample size target.

**Conclusions:**

Successful trial recruitment and conduct in a challenging ED-led trial was driven by trial design, recruitment experience, teamwork and leadership. Our study provides valuable insight from parents and practitioners to inform the design and conduct of future trials in this setting.

**Key messages**

**What is already known on this subject**

- The ‘Emergency treatment with Levetiracetam or Phenytoin in Status Epilepticus in children (EcLiPSE)’ trial was one of the first UK paediatric clinical trials of an interventional medicinal product to be conducted since legislation change enabling research without prior consent (RWPC).
- Pre-trial research identified challenges to the success of EcLiPSE including use of an anti-epileptic medication (levetiracetam), which was not the standard medication in this clinical setting; practitioner anxieties about RWPC and inexperience of conducting an ED-led trial; healthcare staff rotational posts; and availability to seek consent.

**What this study adds**

- In this mixed-methods embedded study to explore parent and practitioner involvement in EcLIPSE.
- Challenges to trial success were overcome through trial design, recruitment experience, parental support for RWPC, and teamwork and leadership.
- Our study provides valuable insight from parents and practitioners to inform the design and conduct of future trials in this setting, including consideration of how the study and RWPC could be briefly communicated to parents of children who are regular ED attenders at the point of randomisation if deemed appropriate.
- Further research is needed to evaluate whether findings and recommendations translate to other ED-led trials of treatments for critically ill children.

## Introduction

Recruitment to multicentre randomised controlled trials is challenging, and poor recruitment can lead to reduced confidence in the results, costly extensions or early closure.^1^ Trial recruitment can be hindered by patient and trial practitioner–related factors including poor quality participant information, practitioner workload pressures, lack of equipoise, lack of leadership, or poor collaboration between clinical specialities and research teams.^2 3^ Trials conducted in paediatric emergency medicine encounter additional practical and ethical challenges, including the need to perform research in life-threatening situations. To help ensure this research can be conducted, clinical trials legislation enables children to be enrolled into some trials without prior informed consent.^4 5^ Research without prior consent (RWPC) is largely acceptable to parents and children, yet they may have concerns if trial interventions are not part of routine clinical care.^6 7^ Studies have also shown how practitioners without experience of RWPC may have negative perceptions of this consent method,^8 9^ particularly if trial interventions represent significant changes to clinical practice.^5^

The ‘Emergency treatment with Levetiracetam or Phenytoin in Status Epilepticus in children (EcLiPSE)’ was an open-label, clinician-led trial and one of the first UK critical care trials of an interventional medicinal product to be conducted since legislation change enabling RWPC. Challenges to the success of EcLiPSE trial were identified in pre-trial research^10^ and site training.^11^ These included use of an anti-epileptic medication (levetiracetam), which was not the standard medication; inexperience of conducting an ED-led trial in a paediatric neurological emergency; practitioner anxieties about RWPC, including how parents would react to trial processes taking place without their consent, such as the opening of a randomisation envelope; healthcare staff rotational posts; and availability to seek consent. Despite these challenges, the EcLiPSE trial successfully recruited to target on time.

EcLiPSE included a mixed-methods embedded study (the Consent Study) involving parents of randomised children and EcLiPSE practitioners to explore experiences of recruitment, RWPC and trial conduct to inform the design and conduct of future ED-led trials. In this manuscript, we explore Consent Study data to identify key obstacles and enablers for successful trial conduct. A framework to enhance practitioner explanations and parental understandings of research without prior consent is reported in a separate manuscript (in press).

## Methods

### Study design, setting and selection of participants

A mixed-methods embedded study (the Consent Study) took place in all 30 EcLiPSE sites between July 2015 and April 2017. This involved questionnaires and interviews with parents of randomised children, interviews and focus groups with EcLiPSE practitioners, and audio-recorded trial discussions. KW (female social scientist, PhD) and LR (female health psychologist, PhD) developed questionnaires (online supplemental file 1), and interview and focus group topic guides, using previous relevant studies^5^ and EcLiPSE feasibility work, which outlined parents’ views on trial acceptability and feasibility, including potential burden of the intervention and approach to consent^10^ (see example questions in online supplemental file 2). Topic guides and questionnaires explored experiences of trial recruitment and consent process, trial acceptability, and perceived barriers and facilitators to trial conduct. Recorded trial discussions between parents and recruiting practitioners enabled additional insight into trial recruitment and RWPC conversations.

10.1136/emermed-2020-209487.supp1 Supplementary data

10.1136/emermed-2020-209487.supp2 Supplementary data

Parents/legal representatives who did and did not consent to their child’s participation in the trial, and all practitioners involved in screening, recruiting, randomising and consenting were eligible to take part. Verbal consent was sought for audio recording trial conversations between parents and recruiting practitioners before study discussions began. If verbal consent was provided, a digital recorder was used to record trial discussions. Written consent was then sought for all Consent Study elements as part of the EcLiPSE consent process. Recruitment for the Consent Study began at the same time sites began recruitment to EcLiPSE. This included written parental consent for the use of recorded trial discussion data, completion of Consent Study questionnaires before hospital discharge and interviews conducted approximately 1 month after enrolment. LR conducted practitioner and parent interviews over the telephone, while practitioner focus groups were conducted face to face in hospital training rooms. Consent for audio recording interviews and focus groups was sought before interviews began. Towards the end of the first year of recruitment, LR identified large and small sites based on attendance numbers, as well as those with high and low EcLiPSE recruitment rates; and contacted lead research nurses or principal investigators to arrange interviews and focus groups. Before interviews and focus groups, LR explained the Consent Study aims and objectives and research processes (eg, consent and confidentiality). As trial recruitment progressed, LR stopped interviewing parents and practitioners from high recruiting sites and purposively sampled across all recruiting sites to ensure sample variance. Interviews and focus groups were audio recorded and transcribed verbatim by a professional transcription company (Voicescript, Bristol, UK). LR anonymised and checked transcripts for accuracy. Qualitative (interviews, focus groups and recorded trial discussion) recruitment stopped when data saturation was reached (the point where no new major themes were identified in ongoing analysis). Questionnaire recruitment took place throughout the EcLiPSE trial recruitment period.

### Data analysis

Our approach to qualitative data analysis was thematic and iterative, referring back and forth between developing analysis, across all quantitative and qualitative datasets for evidence of challenges and enablers to successfully conducting research in emergency situations (see table 1). NVivo software assisted data organisation. LR entered questionnaire data into SPSS. The Consent Study research team (KW, LR) conducted the analysis to ensure analytical rigour. Our approach to synthesising qualitative and quantitative data drew on the constant comparative method.^12 13^ For this analysis, we particularly focused on qualitative and quantitative data to provide insight on enablers and barriers to recruitment and trial conduct. Additional analysis related to practitioner explanations and parental understandings of research without prior consent are reported in a separate manuscript (in press). We present selected interview quotations (with pseudonyms) that illustrate research themes across a range of participants within the results. Where quotes have been shortened for brevity or to remove identifiable information, omitted text is marked with ‘…’ and explanatory text is in brackets. Participants were given consecutive identifying numbers, but cross-checked when presenting quotations for this manuscript to ensure no participant was cited twice with different identifying numbers. Descriptive statistics are presented with percentages.

**Table 1:** Approach to data analysis and synthesis

Phase	Description
1. Familiarising with qualitative data	LR read interview, focus group, audio recorded consent discussion transcripts noting down initial ideas on themes
2. Generating initial codes	Initially, three complementary data-coding frameworks were developed (for focus group, interview and audio-recorded consent discussion data) using broad a priori codes identified from initial reading related to the Consent Study aims and objectives. During the familiarisation stage, LR and KW identified data-driven codes and concepts. Analysis was based on thematic analysis, a method for identifying, analysing and reporting patterns (or themes) within data
3. Developing the coding framework	LR coded three transcripts in each data set and shared the initial coding frameworks with KW. KW second coded transcripts using the initial coding frames and made notes on any new themes identified and how the framework could be refined
4. Defining and naming themes	Following review and reconciliation by LR, revised coding frames were subsequently developed and ordered into themes (nodes) within the *NVivo* Database. Regular meetings were held to discuss the developing frameworks
5. Completion of coding of transcripts	LR completed coding transcripts. For this manuscript, KW reviewed coding specific to challenges and enablers of conducting the trial and conducted further coding across datasets and made notes on in preparation for writing this manuscript
6. Quantitative data analysis	LR entered questionnaire data into SPSS. Descriptive statistics were conducted including χ^2^ test for trend
7. Data synthesis	Our approach to synthesising qualitative and quantitative data^25^ drew on the constant comparative method.^12 13^ This involved KW looking across quantitative and qualitative datasets for themes/data output related to challenges and enablers to successfully conducting research in paediatric emergency situations. This included exploring qualitative themes and quantitative output related to parent and practitioner experiences of recruitment, RWPC and conduct of the trial. Analysis was interpretive—theorising the significance of the patterns and their broader meanings and implications
8. Write-up	LR and KW developed the initial manuscript. KW led the final development of themes and write-up phase in collaboration with LR and MDL.

RWPC, research without prior consent.

### Patient and public involvement

Details of patient and public involvement activities are reported in our linked publication (in press).

## Results

**Figure 1:**
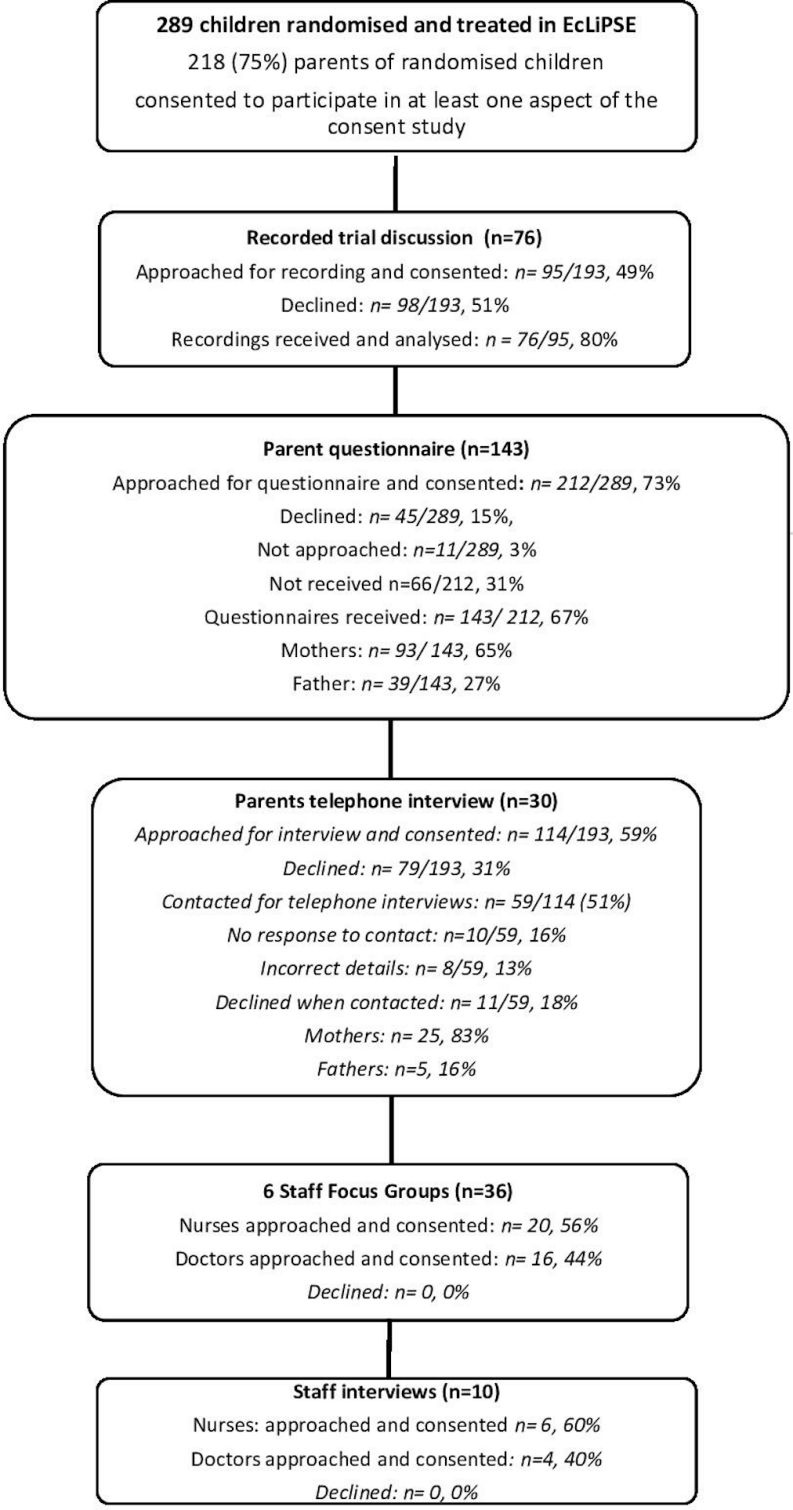
Participant characteristics by method in chronological order.

A total of 218 parents of 289 (75%) randomised and treated children consented to participate in some aspect of the Consent Study, and 143 parents completed a questionnaire (figure 1). We reached data saturation^14^ at 76 recorded trial discussions, 30 parent (25 mothers, 5 fathers) interviews, 6 practitioner focus groups (n=36) and 10 practitioner telephone interviews. All parents interviewed had completed a questionnaire. Practitioners (nurses, n=26; doctors, n=20) included Principal Investigators, Emergency Medicine doctors, and clinical and research nurses. Focus groups were held a mean 12 months and 9 days (range 329–420) after site opening. Telephone interviews took place 5–16 months (mean 8 months and 21 days, range 168–490 days) after site opening. No practitioners in our sample had prior experience of RWPC in paediatric trials.

### Importance of a clinically relevant research question and pragmatic trial design

Practitioners described how the EcLiPSE Study addressed *“a really important question that people want answered*” (Focus group 3, female, doctor P4). This importance appeared to facilitate buy-in and enthusiasm for trial recruitment. Parents also spoke of how the trial aimed to answer a clinical question which they felt was important, with many explaining how they valued research that explores treatments for their child’s condition.

*“I think the importance is I guess the clinical question. I think [this] is when you get maximum engagement from the clinical team”* (Practitioner telephone interview, male, lead research nurse, P9).

*“As the mother of a child that goes into Status and gets severe seizures it’s nice to know that people are looking at studies”* (Recorded trial discussion 41, parent).

Parents whose children had regular seizures described how they saw potential for their child to benefit from trial findings in the future *“if it happened again”* (Parent interview, mother, P28). Our pre-trial feasibility work^10^ had identified inexperience of conducting research during resuscitation as a potential challenge to recruitment. However, during focus groups and interviews, many practitioners described how EcLiPSE trial processes had been easy to follow in an emergency situation. A pragmatic design, which fitted closely to usual clinical practice and did not involve *“extra work”* (Focus Group 2, female, nurse, P4), was valued and appeared to assist practitioner engagement.

## Awareness of randomisation in the ED

An evaluation of EcLiPSE site training^11^ indicated practitioners were concerned that if parents noticed randomisation envelopes being opened in the ED, they would object to the trial, or RWPC. They feared this would cause friction in a highly emotive life-threatening situation.

*“You're like oh my God, what are they [parent] going to say, what are they going to do”* (Practitioner telephone interview, female, lead research nurse, P8).

However, when reflecting on their experience of randomisation, practitioners described how such initial concerns were often not realised as “*parents are focussed on their child”* (Focus group 2, male doctor, P1) and if they had noticed the envelope, it was often a passing recognition. Such findings were supported by the majority of parents who recalled how they “*didn’t really have the time or inclination to think about it”* (Parent interview, mother, P19).

The few parents in our sample who had noticed randomisation taking place in the ED were mainly those with previous experience of their child being admitted to hospital with seizures.

*“When you've got a parent who has a child that’s a known epileptic and so they know all of the normal words that are said around that and then suddenly it’s a different one and they're like what does that mean?*” (Focus group 5, nurse, P6)

In the cases when a parent did notice a change, a brief discussion about the study, including what drug had been allocated, made parents feel that practitioners *“were keeping me involved”* (Parent interview, mother, P15). This communication appeared to alleviate any potential concerns or negative responses.

*“It was after, obviously, the doctor had told me but I had seen them open the envelope, which is obviously new… I think as they opened the letter they would tell me what drug they were using, they always do*” (Parent interview, mother, P7).

Practitioners described how the provision of brief information about EcLiPSE and being “*really transparent*” (Focus group 2, female, nurse, P6) when experienced parents noticed something different was important in maintaining parental trust. However, it was not always possible for practitioners to know whether parents had noticed trial processes were taking place. One parent had noticed the randomisation process, but had not asked questions on noticing the clinical team were whispering about something; this led to parental suspicion:

*“I'm like what’s all this whispering about? What’s the dodgy envelope? My kid is unconscious, tell me what’s going on. So it definitely made me uncomfortable”* (Recorded trial discussion 52, parent).

### Parent and practitioner support for EcLiPSE recruitment and consent processes

Just over one-third of parents (56/143, 39%) who completed a questionnaire indicated they were surprised to find out their child had been enrolled into a trial. However, almost all (139/143; 97%) agreed with the questionnaire statement that they “*understood why consent for my child’s participation in EcLiPSE was sought after the treatment was given”,* while the majority (129/143, 90%) also indicated they were satisfied with the EcLiPSE consent process. In contrast, one mother described her sense of loss of control over the situation as *“They’d done it without telling us”* (Parent interview, mother, P11). However, she explained how she provided consent for the use of her child’s data in EcLiPSE as *“They use these two medications all the time”,* which she found reassuring. As the following quote illustrates, the majority of parents stated that in such a life-threatening situation, parents prioritised the emergency treatment of their child over research consent processes:

*“Well I prefer them to do whatever they can. It doesn’t really matter too much about consent, as long as they can do what they can to stop a seizure or to help stabilise, that is the first and foremost*” (Parent interview, mother P3).

Support for RWPC was also described during practitioner interviews and focus groups, which was mainly attributed to *“really positive responses”* (Practitioner telephone interview, female, doctor, P9) from parents to RWPC discussions.

### Adapting to research: going above and beyond

Evaluation of site initiation training^11^ highlighted concerns surrounding research personnel being available to support clinical teams, leading to patients being missed, or inability to seek consent during evenings, weekends and the busy winter period. Despite best efforts, a minority of sites described how they struggled to work as a team due to lack of research support or engagement by key practitioners.

*“I think not having [nurse] working in research, well that hasn’t helped, I mean just having him there two days that has kind protected time… But even when he was working with us, I don’t think we were necessarily always completing a log screen for every single case”* (Practitioner telephone interview, male, lead research nurse, P9).

However, most sites overcame these challenges and successfully recruited to target, with some sites involved in multiple clinical trials having sufficient research cover at weekends. More commonly, practitioners described how they had *“come in on your time off”* (Practitioner telephone interview, female, doctor, P6). Some high recruiting sites developed methods to maintain trial awareness and ensure eligible patients were not missed, including placing study materials in common areas or resuscitation rooms (eg, posters and leaflets), small non-financial staff incentives, and raising awareness of their recruitment performance in comparison with their own target, and other sites.

### Facilitating trial conduct through leadership

Despite some initial concerns about capacity, site leads acted as advocates for the study, maintaining awareness and regular training:

*“We had taken on the study so we were very keen to do it. Even though we internally had our concerns, we were very strong at promoting it after we had that initial site visit”* (Practitioner telephone interview, female, doctor, P3).

Practitioners emphasised the importance of Principal Investigators taking responsibility for promoting the trial and recruitment of patients 24/7. This was often challenging in EDs working at full capacity. Examples included Principal Investigators preparing resuscitation teams when a potentially eligible child was on their way to the ED in order to briefly refresh people’s minds about the trial and clarify details of the scenario that may unfold.

*“Just, if after the alert, you’d said to whichever nurses are in resus, okay, we’ve got a fitter, we might be doing the EcLiPSE study, are you all aware of that? … What’s going to happen is…”* (Focus group 6, male, doctor, P1).

Strong leadership was not evident at a few sites, which generated negative impact on practitioner engagement, and lower numbers enrolled. Although such examples were rare, they highlight the importance of identifying a motivated Principal Investigator who is willing to make time to support the study throughout trial conduct.

*“Sorry to be a bit miserable about it, but we do struggle a little bit with maintaining any enthusiasm… nobody is taking ownership really”* (Practitioner telephone interview, female, lead research nurse, P1).

## Discussion

This study provides insight into the key factors which enabled successful conduct of a challenging ED-led paediatric emergency care trial drawing on the perspectives of parents of participants, and trial practitioners. Multiple factors, including trial design, tailored communication with parents of regular attenders, research support, teamwork and leadership, contributed strongly to successful conduct at each site and across the trial as a whole. There was a perceived negative impact on team engagement and recruitment where such factors were lacking.

Importantly, both parents and practitioners wanted to know the answer to the research question, a factor that appeared to underpin many of the decisions and behaviours captured in our embedded study. For example, the perceived importance of the clinical question appeared to influence parents’ consent decision as many believed their child may benefit from trial findings in the future. This was not a misconception for parents of eligible children with a chronic health condition, as it was feasible that the trial results could inform changes to clinical care decisions within their lifetime. Our findings also suggested that practitioners were engaged and invested in EcLiPSE due to its design. The trial was open label, pragmatic and clinician led, and aimed to answer an important question that they believed would quickly inform their clinical practice. This engagement was apparent across the majority of EcLiPSE sites, despite initial concerns about whether the trial was possible which were often confounded by inexperience of conducting an ED-led trial. Our findings highlight the important role of researchers and funding panels in identifying clinically important research questions and suggest that challenging trials are more likely to succeed if all key stakeholders, including patients, family members and clinicians, prioritise the research question.^15^

Literature on pragmatic clinical trials has emphasised the importance of understanding the trial context.^16^ ED practitioners valued how the EcLiPSE trial protocol was easy to follow, as it fitted closely to the usual emergency care algorithm for Status Epilepticus. Our findings suggest that work undertaken by the EcLiPSE team, which included ED nurses, doctors, neurologists and triallists, who streamlined intervention delivery and data collection processes to minimise burden, contributed to successful recruitment and conduct in this setting.

As shown in recent pilot studies exploring treatments for paediatric suspected infection,^17 18^ parents and practitioners found RWPC to be acceptable, preventing unethical delays in the delivery of life-saving treatments. Our study adds to this literature by providing further insight into RWPC processes through a mixed-methods approach, including audio-recorded recruitment discussions and interviews with both parents and recruiting practitioners. We found that initial practitioner concerns that parents would notice the randomisation envelope and object to the trial, or RWPC, were most often unfounded. Parents of children with frequent seizures appeared more likely to notice something different was occurring in the ED, such as the opening of the randomisation envelope. These parents valued how practitioners provided them with brief description of the study, including what drug had been allocated. One instance where such communication had not occurred had resulted in parental suspicion and potential breakdown of a trust in practitioners. Practitioners confirmed that brief information sheets and EcLiPSE posters were on display, although few parents noticed or read them. As shown in other studies exploring trial recruitment and decision-making,^19 20^ patients and family members often prioritise verbal over written information provision. The highly stressful and time-critical ED context is likely to have impacted on parental capacity and indeed desire to read even short written study information.^5^ Future trials would benefit from considering how their study and RWPC could be briefly communicated to parents of children who are regular ED attenders at the point of randomisation if deemed appropriate. Box 1 contains suggested brief information used in EcLiPSE practitioner training, which has been adapted for future ED trials.

Box 1 **Communication of RWPC to parents of regular attenders in the ED**

If deemed appropriate, consider explaining how:

- We are conducting a study looking at [add key aim].
- Currently treating your child is the priority. We will of course talk to you about the study as soon as possible after the emergency situation has passed.
- If you would like further information about the study now, we have an information leaflet [direct parent to leaflet and/or posters].

If parents state that they do not want their child included while in the ED, then they should not be included.

Lemiex-Charles and McGuire’s Integrated Team Effectiveness Model (ITEM)^21^ provides a conceptual framework to aid the assessment of teams in healthcare. ITEM outlines the interplay between task (eg, trial) design and processes in achieving team effectiveness, while highlighting the importance of organisational context in which teams are embedded. At some sites, EcLiPSE recruitment was challenged by lack of ringfenced research staff time, or a lack of engagement of key site leads, which led to lower recruitment at these sites. Nevertheless, the majority of sites did not experience or overcome such challenges. Many went above and beyond their expected roles, by working in their own time,^22^ developing internal team rota systems and team incentives to facilitate recruitment. Although we believe this was intrinsically linked to practitioner engagement and support for a pragmatic trial, qualitative data suggest that leadership from the trial team and site Principal Investigators were also contributing factors. Indeed, a lack of leadership at a few sites negatively impacted on practitioner engagement. Practitioners valued regular communication and support from the EcLiPSE team, which included members of the Pediatric Emergency Research in the UK and Ireland (PERUKI) research collaborative.^23^ As shown in other studies,^24^ regular multidisciplinary meetings, including teleconference or annual face-to-face PERUKI meetings, were viewed as a useful method of sharing good practice and maintaining enthusiasm.

### Limitations and future directions

Our embedded study is potentially limited as it relates to only one ED-led trial of treatments for Status Epilepticus in the UK. Further research is needed to evaluate whether findings and recommendations translate to other ED-led trials of treatments for critically ill children. Our study was strengthened by a mixed-methods approach to gain insight into both parent and practitioner experiences of trial recruitment and conduct. All sites participated in the Consent Study and practitioners were purposively sampled for focus groups and interviews to ensure sample variance (eg, low and high recruiting sites). However, LR and KW were members of the EcLiPSE team; therefore, despite confidentiality assurances, their roles may have impacted on practitioners’ willingness to discuss problems with trial recruitment, conduct or record trial discussions with parents. The majority of parents (75%) of children randomised and treated in the EcLiPSE trial consented to participate in some aspect of the Consent Study and qualitative recruitment stopped when data saturation was reached.^14^ However, none of the 19/286 (4%) parents who declined their child’s involvement in EcLiPSE consented to take part in the Consent Study; therefore, their views were not represented.

## Conclusions

A pragmatic trial design, clear communication with parents, teamwork, research nurse support and leadership were key factors in successful recruitment and conduct of a challenging ED-led trial. Our study provides valuable insight from parents and practitioners to inform the design and conduct of future trials in this setting.
