# Supplementary material for: Planning for success: overcoming challenges to recruitment and conduct of an open-label emergency department–led paediatric trial
Source: Emerg Med J. 2020 Oct 13;38(3):191–7. doi: 10.1136/emermed-2020-209487 (PMC7907583; doi:10.1136/emermed-2020-209487)
Supplement: Supplementary data [file emermed-2020-209487supp001.pdf]

Consent study parent questionnaire example questions

1. Please indicate how strongly you agree or disagree with the following statements by placing a circle around the answer that best fits your opinion or decision:

	Agree	Neither Agree nor Disagree	Disagree
a. The doctor or nurse checked that it was a convenient time to discuss research before discussing EcLiPSE	1	2	3
b. The information I received about EcLiPSE was clear and straightforward to understand	1	2	3
c. I understood why consent for the use of my child's information in EcLiPSE was sought	1	2	3
d. I had enough opportunity to ask questions about EcLiPSE	1	2	3
e. I would be happy for my child's information to be used in EcLiPSE **without** being asked (e.g. consent would not be sought)	1	2	3
f. I was satisfied with the consent process for EcLiPSE	1	2	3
g. It was difficult to take in the information I was given about EcLiPSE	1	2	3
h. It was difficult to make a decision about EcLiPSE	1	2	3
i. I made this decision	1	2	3
j. Someone took this decision away from me	1	2	3
k. I was not in control of this decision	1	2	3
l. The decision about the research was inappropriately influenced by others<br><br>If the answer to this question is "Agree" please state who you think influenced the decision about the research:	1	2	3

2. Did you consent for your child's information to be used in EcLiPSE?
Yes [ ] **(Go to question 3)**
No [ ] **(Go to question 4)**

3. What were your reasons for providing consent for your child's information to be used in EcLiPSE? Please tick all that apply AND THEN CIRCLE YOUR MAIN REASON (e.g. ✓)

a. To help my child	
b. To help other children in the future	
c. I felt that medical studies like EcLiPSE are important	
d. Because I trusted the doctor or nurse who explained EcLiPSE	
e. My child's seizure stopped	
f. I didn't feel comfortable saying no to the nurse or doctor who explained the study	
g. Other (Please state):	

4. If you did not provide consent, please provide your reasons for deciding that your child's information would not be used in the EcLiPSE study (If you do not wish to do so please leave this space blank):

5. We would value any comments or suggestions you have to improve the recruitment and consent process for EcLiPSE, please use the space below:
